# Explaining the Process of Spiritual healing of Critically-ill Patients: A Grounded Theory Study

**DOI:** 10.4314/ejhs.v30i4.13

**Published:** 2020-07-01

**Authors:** Forough Rafii, Mahmoud Eisavi, Mehdi Safarabadi

**Affiliations:** 1 Nursing Care Research Center (NCRC), School of Nursing and Midwifery, Iran University of Medical Sciences, Tehran, Iran; 2 Departement of Islamic Economy, Allameh Tabatai University, Tehran, Iran

**Keywords:** Spiritual Therapies, Spirituality, Refractory disease, Grounded Theory

## Abstract

**Background:**

Spiritual healing is one of the most intriguing category of alternative and complementary medicine. The aim of this study was to explain the process of spiritual healing in patients with refractory diseases in Iran.

**Methods:**

This grounded theory study was conducted in Iran from 2018 to 2019. The participants were 14 patients with refractory diseases and 4 healers whom were first selected through purposeful and then theoretical sampling. Semi-structured interviews were used to collect data on patients and healers. All the interviews were transcribed verbatim. Data were coded and grouped under specific categories and analyzed using the Strauss and Corbin's approach (2008).

**Results:**

Four main categories emerged from data analysis including: I) frustration to initial acceptance II) disbelief to trust III) evaluation to action and IV) doubt to certainty.

**Conclusion:**

The results of our study provide context-specific factors affecting the complex and multifactorial nature of spiritual healing process in patients with refractory diseases. Health care professional can use these findings in designing and implementing appropriate interventions to integrate spiritual healing into their holistic practices of care.

## Introduction

Over the last fifty years, nursing care has made impressive progress towards a comprehensive care (1). In the holistic care model, human is regarded as a biological, social, psychological, and spiritual being; all aspects of his being are taken into consideration in the care process. Because these dimensions are dynamic and interrelated, it is necessary to pay more attention to all of them for their potential health benefits (2). The results of previous studies suggest that among these dimensions, the spiritual one received relatively less attention (3). Nonetheless, the spiritual dimension encompasses other aspects of human existence and can be defined as a coherent dimension in the well-being and health of each individual. In times of crisis and disease, spirituality becomes more important, and spiritual needs become more apparent (4). On the other hand, during the illness process, spiritual coping strategies is frequently used by patients. Spiritual healing and praying are among the most widely used spiritual sources by the patients (5).

Spiritual healing is one of the most intriguing category of alternative and complementary medicine. Spiritual healing is a systematic and purposeful intervention performed by one or more people to help another person, focused on improving their conditions (6–9). There is evidence that the use of spiritual healing has been increasing rapidly over time, worldwide. Over 64.1% of Americans take advantage of prayer and spiritual healing. The word “spiritual healing” is a generic term that encompasses a range of methods (10–11). Healing prayer is one the categories of spiritual healing. In healing prayer, the healer does not have any contact with the patient's body; also called distant healing or distant healing prayer. Spiritual healing through healing prayer often involves healers. Healers are those who perform non-medical treatment by hands or from distance (12–14). Spiritual healing through prayer has at least two components as the recipient; someone with a physical or mental illness and the healer; one who uses the power of faith and prayer to heal (3). In Iran, some patients refer healers to pray for their healing (13). The healers claim that they can complete cure for the patients' (recipient's) disease through healing prayer (11).

There is ample evidence suggesting that some patients with refractory diseases, for whom the medical interventions have had no permanent positive results, were likely to benefit from healing prayer; although the exact mechanism by which the prayer exerts its effect is not fully elucidated (15). Additionally, the researcher's experience supports the little existing literature which some patients with serious health problems - who have not been able to achieve the improvement through medical interventions have shown clinical improvement by referring to some healers and asking them to pray for their illness.

Considering the increasing trend in using complementary and alternative modalities by patients, especially patients with refractory or incurable diseases, and the paucity of qualitative research evidence addressing the process of spiritual healing in patients with refractory diseases, the aim of this study was to explore the spiritual healing process in patients with refractory diseases through a grounded theory study.

## Methods

In this study the grounded theory methodology was used due to its strength in explicating human behavior in context. According to Corbin and Strauss (2008) grounded theory is a qualitative research methodology used to investigate the social processes involved in human interactions, and the structure and the process leading to them (16–17).

### Participants:

Eighteen participants, including 14 patients with any refractory diseases and 4 healers, were selected and participated in this study from 2018 to 2019. To better understand the process of spiritual healing, the participants were sought to represent a wide range of individuals in terms of work experience, duration of illness, level of education and beliefs. The sampling was started with purposeful sampling. The inclusion criteria for healers were familiarity with prayers and the evidence indicating the positive effects of their prayers on the patients' physical or mental illness reported by the healers themselves or the patients (along with clinical tests before and after the illness). Also, the inclusion criteria for the patients were being over 18, having religious faith, having a history of a confirmed refractory disease not treated completely by medical treatments and eliminated completely after the healing proved by a medical certificate, and having enough time and willingness to participate in the study. Exclusion criterion was the unwillingness of the participant to continue the study.

### Data Collection:

Data were gathered using semi-structured individual interview. This study was conducted in Iran, between 2018 and 2019. The interviews were conducted at the participants' homes. The sample size was defined through theoretical saturation. . To begin each interview, the place of interview was chosen in a way to be quiet and comfortable for the interviewee. The participants were informed in advance of the approximate duration of the interview. The interview with the patient started with an open-ended question such as “*Please tell us about the moment when you found out you had a disease that its treatment is difficult*”. Similarly, the healer interview was started with the question like “P*lease tell us about the time when a patient with a refractory disease comes to you*”. These open-ended questions were developed to stimulate conversation and encouraging the participants to openly share their personal experiences. Also, the probing questions such as “*Could you tell me more about that*” were used to clarify some presented concepts by participants. Duration of the interviews varied from 54 to 108 minutes.

### Data analyses:

Interviews were audio-taped for accuracy and verbatim transcription. After the first interview, data analysis was performed manually in WORD software, immediately after transcription verbatim of each interview. The authors reviewed the transcripts several times to get insight into the participants' experiences. Using continuous comparison analysis, data were coded, and related codes were finally grouped under certain categories. Data analysis consists of four steps: analyzing data for concepts, data analysis for context, bringing process into the analysis, and integration of categories. It is worth noting that these four steps of analysis are not linear and often occur simultaneously (18–19).

### Rigor:

The criteria proposed by Lincoln and Cuba (20) were used to establish trustworthiness of the study findings. Member checking, integrating the data sources and method integration, prolonged engagement, transcribing the interviews as soon as possible and peer debriefing were used to ensure and increase data trustworthiness. In addition, the researcher carefully registered the research documentations to allow an external reviewer to evaluate the study.

### Ethical Considerations:

Approval of institutional Research Ethics Committee of Iran University of Medical Sciences (code: IR.IUMS.REC.1397.1058) as well as participants informed consents were obtained.

The participants were assured of the confidentiality of the data and their right to participate in the study or to leave it at any time and for any reason.

## Results

Demographic and clinical characteristics of the participants are presented in Table 1. The analysis of the data resulted in emergence of the four main categories: I) frustration to initial acceptance, II) disbelief to trust III) evaluation to action and IV) doubt to certainty. Also, 12 subcategories, which each of them explaining a part of the overall process of spiritual healing in patients, have been emerged (Table 2).

**Table 1:** Demographic characteristics of the participants

Participant	M/F	Age (year)	Education	Duration of illness (year)	Reference to spiritual healing	Time past spiritual healing (year)	Type of disease
1	F	26	PhD	2	Family/friends	3	Schizophrenia
2	F	38	Diploma	7	Mother	5	Thalassemia Major
3	M	53	Master's degree	1	Brother-in-law	3	Renal Tumor
4	F	54	Diploma	2	Daughter	2	Liver Cancer
5	F	50	Below diploma	1	Friend	1	Renal Tumor
6	M	25	Diploma	4	Sister	4	Major Depression
7	M	21	Diploma	3	Father	3	Multiple Sclerosis
8	F	25	Bachelor's degree	1	Friends	1	Brain Tumor
9	M	30	Master's degree	2	Friends	3	Depression
10	F	34	Bachelor's degree	4	Brother	3	Cardiac Disease
11	F	30	Bachelor's degree	2	Spouse	5	Bipolar Disorder
12	M	45	Below diploma	2	Friends	2	Kidney Transplant
13	M	60	Below diploma	1	Child	1	Lung Cancer
14	F	28	Diploma	8	Spouse	3	Infertility
**Healer**	**M/F**	**Age (year)**	**Education**	**Period of practicing healing (year)**		**Number of patients (per month)**	
1	M	55	PhD	30		10	
2	M	40	Master's degree	10		2	
3	M	38	Bachelor's degree	7		1	
4	M	46	Bachelor's degree	17		8	

M: male; F: female

**Table 2:** The process of emergence of categories and subcategories and primary categories of patients' spiritual healing

Frustration to initial acceptance	Frustration with medical treatment	Discontinuing medical treatment
		Despair (frustration)
	Being in limbo	Confusion after leaving discontinuing medical treatment
		Suicidal thoughts and imminent death
	Seeking non-medical ways	Introduction to prayer writing
		Herbal
	Being selected	Through identification of patient
		Through those familiar with spiritual healing
Disbelief to trust	Initial distrust	Initial total rejection of spiritual healing
		Comparing spiritual healing with other methods
	First impression	Through the healer's spirituality
		Patient's trust in spiritual healing
		The attraction of the meeting place
		Hope created by healer
Evaluation to action	Evaluation	Initial investigation
		Psychosocial evaluation
		Mental evaluation
		Spiritual evaluation
	Prescriptions	Prescription of religious duties by the healer
		Prescription of sin avoidance by the healer
		Prescription of spiritual prayers by the healer
	Action	Distant prayer by the healer
		Performing religious duties by the patient
		Avoidance of sin by the patient
		Performing spiritual prayers by the patient
Doubt to certainty	Confronting doubt	Temporary frustration and doubt
		Overcoming the doubt by the patient
		Saying spiritual prayers
	Second meeting	Reasons for the second meeting
		Spiritual instructions
	Gradual healing	Mental & psychological changes coinciding the healer's praying
		Physical changes coinciding the healer's praying
		Spiritual changes coinciding the healer's praying
		Complete healing

### Frustration to Initial Acceptance:

This category includes frustration with medical treatments, being in limbo, seeking non-medical ways and being selected. Patients at this stage are frustrated due to lack of benefit from medical treatment, following progression of the disease and uncertain benefits of treatment. So, they look for non-medical interventions to dealing with their disease, such as herbal remedies, but do not achieve the desired results. Following this helplessness and uncertainty, the patient becomes acquainted with spiritual healing, making them to be selected by the healers for spiritual healing.

At the beginning of this phase, patients become completely disappointed in the medical treatment. For example, a participant mentioned that: “*In the early days of my illness, I did a number of things and I liked to see the doctor and take the medicines. But it took so long and I didn't get the desired answer and my illness became worse, so I had no hope of curing my illness*”.

Being in limbo was the next step and the patients were forlorn and helpless that the medical treatment will not be effective. For example a participant mentioned that: “*After returning from the doctor's office, I decided not to see the doctor at all; I didn't even want to take my medications anymore, because I was confused and bored as I was getting worse. I was torn between death and life*”.

After this stage, the patients were frustrated with the treatment but were looking for other nonmedical interventions, such as witchcraft and herbal remedies. A participant stated that: “*I didn't believe in magic, but my family decided to take me to try a witchcraft, I had no idea to make a decision because I was in a terrible situation and they took me*”. Another participant mentioned that: “*My daughter offered herbal remedies, but it is not the treatment for this diseaseas the chemical medicines had not worked*”.

### Disbelief to Trust:

This category includes early distrust and first impression. At the beginning of the patient's acquaintance with spiritual healing, they develop an initial distrust to this method that causes them to completely reject it. A participant stated that: “*When my brother told me that some patients are cured in this way, I didn't believe it and I wondered how it was possible*”. This initial distrust to spiritual healing made patients immediately compare spiritual healing to other therapies. A participant mentioned that: “I *said that I couldn't be treated by doctors, so how could I be healed by healers?*”

However, when the patients came to the healers, they experienced a positive first impression. This first impression was partly due to the healers' spiritual mood and relative insignificance of financial matters to them. Following this impression, the patients found a glimmer of hope for the treatment of their illness. A participant said: “*He didn't ask for money. He was a very spiritual man. I received some kind of energy from him and I felt that God would answer his prayers and I trusted him a little*”.

On the other hand, this first impression was due to the spiritual atmosphere of the meeting place, which healers preferred it to be a sacred place. A healer mentioned that: “*I prefer to meet the patients in a sacred spiritual place, because such places have positive energy*”.

### Evaluation to Action:

This category included an evaluation, prescriptions and an action. At this stage, healer-patient interactions are at their peak. In the evaluation phase, the healer examines the patient's potential and capability for performing religious practices through an initial examination. On the other hand, by evaluating the physical, mental and spiritual dimensions of the disease, he tries to understand the relationship between the disease and these dimensions. Completing the evaluation, the healer begins spiritual prescriptions for the patients, and performs the distant prayer for them. Immediately after the prescription of spiritual instructions, the patient does them in absolute belief.

The initial evaluation begins with an assessment of the patient's spiritual potential and the course of their illness. A healer said: “*When I see the patient closely, I evaluate their energy for saying the prescribed spiritual prayers to see if they have the required capability or potential*”. The healers also ask some questions about the course of illness. Another healer mentioned that: “*When the patient comes to us, we start asking questions. We ask about their illness, condition and all the actions and measures they have taken, for example, about the doctors they have seen and what they have done*”. Also, the healer begins to assess the patient's psychological and social status. He examines this dimension by asking questions about the patient's mental and social status. The healer also evaluates the patient by asking some questions about the physical dimension. However, the most important dimension that the healer evaluates is the spiritual dimension of the patient. The healer evaluates this dimension by asking questions about the religious practices performed by the patient, their communication with God and their beliefs. A participant mentioned that: “*He (healer) put a lot of emphasis on believing in God and said that healing this way requires believing in God. The healer kept asking me some questions about prayer and fasting, and he would say I couldn't do anything for you if you had no connection with God*”. After this stage, the healer performed spiritual prescriptions for the patient. At this point, the emphasis is on performing the religious duties and forsaking the sins by the patient. A participant states that: “*The healer said “You must be bound to pray, not to talk behind someone's back, not to slander and not to sin in general*”.

After that, healers determine specific spiritual prayers for the patient to say during a certain period of time. These prayers root in the Quran and the words of the infallible Imams. The healers emphasize that the patient must say the spiritual prayers during a specified period of time. A participant said: "He told me to recite Surah Hamd of the Quran seven times after each prayer and whenever I had the pain, I would repeat this prayer: “there is no power except in God Almighty”.

### Doubt to Certainty:

This category included exposure to doubt, second meeting and gradual healing. The patients sometimes had a temporary doubt in spiritual healing during the execution of spiritual instructions and asked themselves whether this treatment was false? This doubt arises in times of despair of spiritual healing or progression of symptoms in patients. A participant mentioned that: “*At the beginning, one day I was feeling bad again. Suddenly I asked myself “Am I going the wrong way? Is it all a lie?” Then, my pain got worse*”. In such cases, the patients communicate the problem with the healer through a phone call. The healers try to persuade them to perform the spiritual instructions by giving comfort to them, distant praying and prescribing the continuation of spiritual instructions.

## Discussion

The results of this study showed that patients with refractory diseases become frustrated with medical treatment over time. They usually facing the severity of their diseases and the lack of effective treatments that eventually leads them to use of alternative modalities. The results of the qualitative study of Barlow et al. showed that the patients with breast cancer undergoing hormone therapy for a long time try complementary and alternative therapies due to lack of appropriate therapeutic response (21). In the present study, the patients are looking for a way to cure their refractory disease. A number of studies show that the patients who are seriously-ill and have failed to manage their illness are seeking a way to have potential capacity to cure their disease. As a result, they have chosen to use traditional and complementary healing modalities (22). These findings are consistent with the results of our study. In the next stage, the patients are introduced to the spiritual healing by those who have experienced it or are familiar with it and are selected by the healers. Muslims in Iran believe that humans are helpless against the smallest pathogen, such a Virus, and despite all the mounting research, they continue to be ignorant about the cause of cancer and many other debilitating diseases (14).

The results of a qualitative study by Al-Mutair et al. indicated that critically-ill patients are encouraged by their family members to try the spiritual healing and they refer to the healers (23). These results are also consistent with the results of the present study. Another category of this study was disbelief to trust. Most people choose medical treatment facing an illness (24). However, the usage rate of complementary therapies among people is increasing. For example, over 64.1% of Americans enjoy spiritual healing and praying (25). But all the dimensions of spiritual healing and healing prayer have not yet been identified and the patients are not familiar enough with them and may not accept them (26). In illness, the awareness of God increases and Muslims become closer to God by realizing their own weakness (27).

In the present study, healers tried to meet patients in a spiritual setting. The patients also reported that as soon as they entered the meeting room, they would experience positive mental, physical and spiritual changes. In a study by Teut et al, the patients experienced physical and mental changes when they meet the healers and their symptoms were alleviated so that they reported they received some kind of energy when they were with the healers (28). These results are consistent with those of the present study that patients have positive feelings in spiritual places. This trust in the healer and positive effects of the meeting place leads to first positive effects on the patients. In a systematic review by Astin et al. the positive effect of healers on patients has been confirmed. This result is consistent with those of the current study (29).

Another category of this study was evaluation to action. It includes the subcategories of evaluation, prescription and action. The healers evaluate the patients after attending the meeting. This evaluation covers some questions about the patient's physical, psychological and spiritual status so as to examine the dimensions of the patient and provide them with appropriate spiritual prescriptions and pray for the patient form a distance. The healers did not pay attention to clinical tests and patients' symptoms. Tatsumura et al. indicates that the healers do not diagnose the disease and do not prescribe medical medicines, but they claim that they connect to the main source of energy (God) and recover the balance of the body and help the human to reach their physical, mental and spiritual totality (30). The worldview of Muslim patients towards health and illness incorporates the notion of receiving illness and death with patience, meditation and prayers. Muslim patients understand that illness, suffering and dying are part of life and a test from God. Health and illness become part of the continuum of being, and prayer remains the salvation in both health and sickness. It is narrated that the Prophet Mohammad said that: “the prayer of the sick person will never be rejected, until he recovers”. Finally, this reduces symptoms of the disease, accelerates the recovery and motivates the patient and brings about wellbeing and calmness (31). These results are consistent with those of the present study.

Healers prescribe spiritual prescriptions such as prayer, reciting Quran and special prayer for patients in specific period of time. Then, the patients perform the spiritual instructions. Since the beginning of Islam, religious practices, such as prayer, have been an alternative to medical treatment and they are still a common practice in the Islamic world. The Muslims invite others to use the Quran's prayers for healing and being healthy (32). The Muslims pray in times of crisis and even in the Shia School of thought, patients receive healing by resorting to infallible Imams (14). The word “prayer” has been mentioned 200 times in the Quran for people to ask God and pray for their problems (33–34). The last category of this study was doubt to certainty. It included subcategories of initial doubt, second meeting and gradual healing. In this study, the patients experienced doubt toward the spiritual healing during the healing period whenever the symptoms became worse and when they were disappointed. Cancer and incurable diseases are among the most leading causes of mortality. Due to the fear of death and the anxiety and stress related to it, mental and psychological disorders and discomfort are common in these patients, such as frustration, disappointment, instability and reduced social energy (35). Insufficient understanding of pray and God by humans can strengthen the stress and doubt and take them far away from the spiritual line (36). Accordingly, the patients in this study experience doubt at the beginning of the spiritual healing. The results are consistent with those of the present study.

In this study, distant praying by healers for the patients led to the patients' gradual healing. On the other hand, Javaheri et al. described the experiences of the healed patients through healing prayer in a qualitative study and points out that hope, happiness, calmness, positive feelings and positive results are gradually appeared during the multiple sessions (13). The results are consistent with those of the present study.

In conclusion, the results of our study provide context-specific factors affecting the complex and multifactorial nature of spiritual healing process in patients with refractory or incurable diseases. Health care professional can use the findings of this study in designing and implementing appropriate interventions to integrate spiritual healing into their holistic practice of cares.
